# The Frontiers of Functionalized Nanocellulose-Based Composites and Their Application as Chemical Sensors

**DOI:** 10.3390/polym14204461

**Published:** 2022-10-21

**Authors:** Mohd Nor Faiz Norrrahim, Victor Feizal Knight, Norizan Mohd Nurazzi, Mohd Azwan Jenol, Muhammad Syukri Mohamad Misenan, Nurjahirah Janudin, Noor Azilah Mohd Kasim, Muhammad Faizan A. Shukor, Rushdan Ahmad Ilyas, Muhammad Rizal Muhammad Asyraf, Jesuarockiam Naveen

**Affiliations:** 1 Research Centre for Chemical Defence, Universiti Pertahanan Nasional Malaysia, Kem Perdana Sungai Besi, Kuala Lumpur 57000, Malaysia; 2 Bioresource Technology Division, School of Industrial Technology, Universiti Sains Malaysia, Penang 11800, Malaysia; 3 Green Biopolymer, Coatings & Packaging Cluster, School of Industrial Technology, Universiti Sains Malaysia, Penang 11800, Malaysia; 4 Department of Bioprocess Technology, Faculty of Biotechnology and Biomolecular Sciences, Universiti Putra Malaysia, Serdang 43400, Malaysia; 5 Department of Chemistry, College of Arts and Science, Yildiz Technical University, Esenler, Istanbul 34220, Turkey; 6 Department of Chemistry and Biology, Centre for Defence Foundation Studies, Universiti Pertahanan Nasional Malaysia, Kem Perdana Sungai Besi, Kuala Lumpur 57000, Malaysia; 7 School of Chemical and Energy Engineering, Faculty of Engineering, Universiti Teknologi Malaysia, Johor Bahru 81310, Malaysia; 8 Centre for Advanced Composite Materials (CACM), Universiti Teknologi Malaysia, Johor Bahru 81310, Malaysia; 9 Engineering Design Research Group (EDRG), School of Mechanical Engineering, Faculty of Engineering, Universiti Teknologi Malaysia, Johor Bahru 81310, Malaysia; 10 School of Mechanical Engineering, Vellore Institute of Technology, Vellore 632014, India

**Keywords:** nanocellulose, composites, chemical sensors, functionalization

## Abstract

Chemical sensors are a rapidly developing technology that has received much attention in diverse industries such as military, medicine, environmental surveillance, automotive power and mobility, food manufacturing, infrastructure construction, product packaging and many more. The mass production of low-cost devices and components for use as chemical sensors is a major driving force for improvements in each of these industries. Recently, studies have found that using renewable and eco-friendly materials would be advantageous for both manufacturers and consumers. Thus, nanotechnology has led to the investigation of nanocellulose, an emerging and desirable bio-material for use as a chemical sensor. The inherent properties of nanocellulose, its high tensile strength, large specific surface area and good porous structure have many advantages in its use as a composite material for chemical sensors, intended to decrease response time by minimizing barriers to mass transport between an analyte and the immobilized indicator in the sensor. Besides which, the piezoelectric effect from aligned fibers in nanocellulose composites is beneficial for application in chemical sensors. Therefore, this review presents a discussion on recent progress and achievements made in the area of nanocellulose composites for chemical sensing applications. Important aspects regarding the preparation of nanocellulose composites using different functionalization with other compounds are also critically discussed in this review.

## 1. Introduction

**Figure 1:**
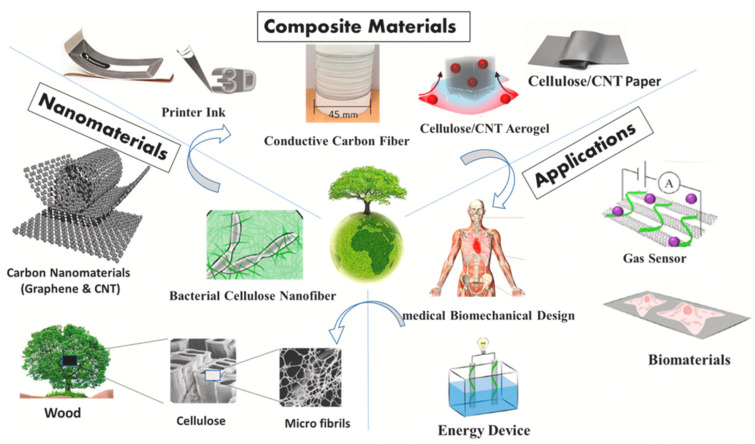
Polymer composite materials and its various applications. Adapted from Ref. [9].

Recent developments in material science have seen a new class of materials known as “mixed materials” or “composites” emerging [1,2,3,4]. Polymer composite materials are materials that are made up of organic semiconductors and biomass derivatives that could be used in a variety of ways [5]. These combinations provide a material with improved mechanical strength, electrical conductivity and thermal stability [6,7,8]. Figure 1 illustrates composite materials used in a variety of different applications. 

Nowadays, polymer composites are being used more commonly in the development of chemical sensors [10,11,12,13]. Chemical sensors have gained significant attention in the various areas of public safety, such as in the military [14], space exploration [15], biomedicine [16], pharmaceuticals [17], leakage detection of explosive gases such as hydrogen [18], and real-time detection of toxic and carcinogenic gases in various industries [19,20], as well as chemical warfare agents [21,22], particularly at public venues such as airports and public parks. These chemical sensors are typically installed both indoors and outdoors, as well as used as portable devices to be brought into areas where the target analytes are suspected or spilled. The need for more accurate and sensitive chemical sensors has driven research and development of these sensors with the use of polymers and composites now being given more emphasis most likely due to the remarkable sensor performance resulting from their use. This improved sensor performance is increased sensitivity in the parts per million (ppm) to billion (ppb) range for trace level detection, absolute discrimination, and the sensors now becoming bio-based, reproducible, biocompatible, with the ability to operate at mild operational temperatures, having low power consumption, of a reasonable size, volume and mass, and with low cost for large-scale applications [23,24]. However, the development of the ideal chemical sensor is still far from realisation in spite of the enormous advances over the past few decades.

Recently, as various material science fields have expanded, there has been a surge in interest in further improving dependable graphene composite chemical sensors. Interestingly, nanomaterials are being actively investigated in the ongoing development of composites for chemical sensors. This is because of these nanomaterials having extraordinary physicochemical properties that are absent in their bulk form [25,26]. Hence, over the last few decades, nanomaterials have been actively investigated and then applied as core components of advantageous chemical sensing applications. 

**Figure 2:**
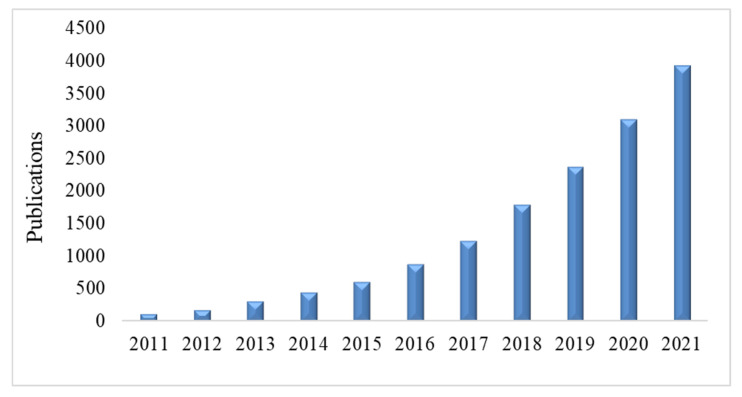
Total number of publications related to nanocellulose chemical sensors.

Composites can be derived from plant-based nanomaterials, among which is nanocellulose which has attracted significant attention as potential replacements for their more conventional petroleum-derived counterparts for use in chemical sensing applications. The current trend seen in publications related to this area show an extensive increase over this past decade. This is illustrated by a survey using Google Scholar using the keyword “nanocellulose composites for chemical sensors” which is shown in Figure 2. Nanocellulose possesses several interesting properties which includes being a renewable resource, having a large specific surface area, a porous structure, essentially biocompatible and with unique structural and physical characteristics such as tensile, optical and electrical properties which make it an ideal material for use in chemical sensors [14]. However, since the natural hydrophilic property of nanocellulose is not compatible with the hydrophobic nature of some sensing molecules, the development of suitable composites using surface functionalization on the nanocellulose is required to enable the needed sensor component compatibility. 

Nanocellulose composites are usually prepared by functionalizing them with a variety of conducting polymers such as polypyrrole (PPy), polyaniline (PANI) and poly (3,4-ethylenedioxythiophene) (PEDOT) derivatives [27,28,29,30]. This is to develop a sensor mediating material that has the necessary electronic characteristics together with the structural advantages that nanocellulose has. Conducting polymer nanostructures with large specific surface areas and a porous structure that are combined with suitable electrical properties have been reported to be excellent sensing mediators [31].

This review article provides a state-of-the-art review on the performance of nanocellulose composite applications as chemical sensors. It not only covers the fundamental aspects regarding the capability of nanocellulose as chemical sensors but also several important strategies such as functionalization and hybridization of nanocellulose composites for chemical sensor applications. Their mechanisms with regards to specific gases were also highlighted. This review concludes by presenting an overview of the challenges faced and recommendations for future advances.

## 2. An Introduction to Chemical Sensors

A chemical sensor is a self-contained instrument that can offer real-time analytical information on the presence of chemical species in a particular environment [32]. Transduction and identification are two roles that a chemical sensor performs. Initially, an analyte has a selective interaction with the identification element. Identification receptors are different molecular entities that make up the sensing element [33]. The ion sensor was the first type of chemical sensor, and it has been extensively developed and widely utilized [34]. The glass pH electrode is the most widely used ion sensor today. This type of sensor was invented by Klemensiewicz and Haber in 1908, and it became commercially accessible in 1936, along with the Beckman pH meter [35].

In recent times, the emissions of toxic by-products from human activity and as pollutants, such as nitrogen oxides (NO_x_), carbon oxides (CO_x_), sulphur oxides (SO_x_) and ammonia (NH_3_), have considerably increased, now posing a long-term threat to our health and the environment. Thus, chemical sensing devices have been extensively developed and studied to monitor those chemicals that are detrimental to human health and the environment. Improvements and optimizations of existing chemical sensors, including gas sensors, together with the development of new sensors with higher sensing performance and sensitivity at a lower cost, continue to be needed for a variety of applications, which go beyond industrial sectors but also include indoor health and safety, environmental monitoring, and many others [36].

Chemical sensor development is undergoing several new and continuing developments. The phrase “nanoscience is revolutionizing a field” has since become a cliché but it does play an important part in chemical sensor development. Swager and Mirica (2019) [37] claim that the tremendous innovations of the recent decades have had a profound impact on the sensor industries, where the analyte transduction interface just needs to be made nanoscale. Materials can be manufactured or examined via nanofabrication, use sophisticated imaging technologies with sub nanometer resolution, and sensor developers have access to nanomaterials generated from the bottom up or top down. Nanostructured materials are being used to push the limits of a new generation of sensors using these nanomaterials, which includes metal oxides, thus taking these newly developed sensors to a higher level than that achievable at present.

Metal oxide nanostructures such as titanium oxide (TiO_2_), iron oxide (Fe_2_O_3_), zinc oxide (ZnO), stannous oxide (SnO_2_), tungsten oxide (WO_3_), cuprous oxide (Cu_2_O) and others similar, have been studied extensively for use in sensing applications. This is because of certain characteristics, including a large specific surface area, superior mechanical flexibility, strong chemical stability and improved sensitivity [38,39]. Nonetheless, these metal oxide-based sensor materials, on the other hand, have significant restrictions, such as needing a high operating temperature (100–500 °C), thus resulting in high power consumption which then negatively impacts integration and long-term stability of the sensor device. Over the past few decades, metal oxide gas sensors have been used to detect certain gas species at high working temperatures, which are required to stimulate gas reaction with oxygen ionosorbed over the semiconductor, causing a shift in the material’s resistance. In actual usage, the high-temperature operations needed for the function of these sensors may increase the risk of fuel ignition while used to sense the presence of high explosive gases. When hydrogen is mixed with air, the presence of oxygen at a concentration of 4% (at the lower explosive limit), this mixture can explode. Hence, the fabrication of sensors that can operate efficiently at room temperature is vital [40].

Aside from that, most commercial gas sensors are made from semiconductor and polymer materials, and the sensing methods available include optical, chemiresistive, calorimetric, gas chromatographic, and acoustic [41]. These gas sensors typically have one or more of the following limitations: high power consumption, high cost of operations, poor sensitivity at parts per billion (ppb) level, poor selectivity, limited life span, poor repeatability, and downsizing the sensor devices is very challenging [42].

In contrast, nanomaterial gas-sensing materials have gained a lot of traction as an alternative because of many promising electrical, optical, and thermal properties, as well as their high surface to volume ratio, short response and recovery times, high sensitivity, selectivity, reversibility and stability properties [43]. Other than metal oxides, nanocellulose shall be discussed in this review as it has been shown to be useful in the development of composites for chemical sensors due to its relative ease in tailoring its sensitivity through functionalization [44]. 

Sensors which utilize nanocellulose can be divided into the following types: optical, acoustic wave-based, piezoelectric and electrochemical [45]. Optical sensors can be based on fluorescence or surface-enhanced Raman spectroscopy sensors. An example of a sensor based on acoustic waves is an ammonia sensor that uses a quartz glass microbalance with a sensing coating composed of negatively charged electrospun cellulose acetate nanofiber [46]. Piezoelectric nanocellulose/graphene sensors have been fabricated for use in strain sensing while electrochemical sensors based on chemically modified nanocellulose have been designed in the most part for detecting pathogenic bacteria and glucose, cholesterol, and organic liquids [47]. 

Recently, there have been reports regarding nanocellulose as the sensor material including gas sensor, chemical sensors, biosensor, heavy metal ion sensing, temperature sensor and strain sensor [48,49,50,51,52]. Since the nanocellulose has a large surface area [53] and is rich with the hydroxyl group [54], it can be functionalized with other chemicals depending on the targeted analytes. Therefore, more focus was given in this review on the functionalization on nanocellulose and its mechanism as chemical sensor materials. 

## 3. Nanocellulose’s Unique Characteristics as a Chemical Sensor 

**Figure 3:**
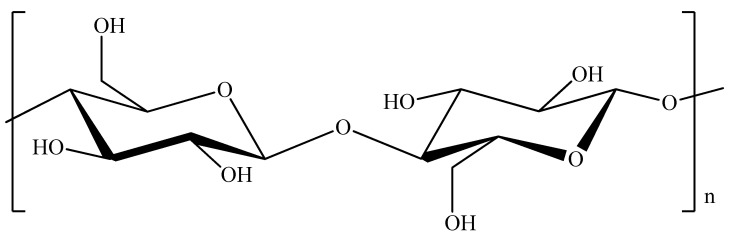
Chemical structure of cellulose.

Natural cellulose constitutes the most abundant renewable polymer resource available worldwide [55,56,57]. As a raw material, it is generally well known for its use in the form of fibers or derivatives in a wide spectrum of products and materials for a multitude of uses. Cellulose fibrils are structural entities formed through a cellular manufacturing process, involving cellulose biogenesis, and stabilized by both hydrogen bonds and van der Waal forces. It contains an abundance of hydroxyl groups as shown in Figure 3. It can be further converted into nanocellulose through several pre-treatment approaches involving chemical, mechanical, physical, and enzymatical processes or through combination of processes thereof [58,59,60,61].

**Figure 4:**
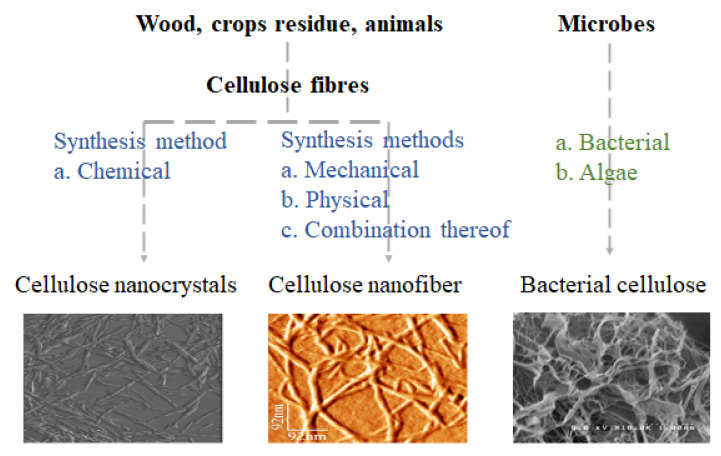
Morphology of cellulose nanocrystals, cellulose nanofibers and bacterial nanocellulose. Adapted from Ref. [14].

Nanocellulose can be classified variously into cellulose nanocrystals (CNC), cellulose nanofibrils (CNF) and bacterial nanocellulose (BNC) depending on their production mode as well as their morphologies. These are presented in Figure 4. CNC are long and straight crystals of cellulose with very large modulus elasticity and strength. CNC are needle-shaped crystalline fibrils that are 150–300 nm in length and 5–10 nm in diameter. They are mainly produced by the controlled acid hydrolysis of cellulose fibers which selectively dissolves the amorphous domains and releases cellulose crystallites. Meanwhile, CNF is composed of thin, flexible nanosized fibrils, encompassing both crystalline and amorphous domains. Their production involves mainly intensive mechanical disintegration. This is done using either high pressure homogenization, microfluidization or high-intensity mechanical grinding using wet disk milling [62,63,64,65]. On the other hand, BNC is made of cellulose nanofibrils which are obtained from certain types of bacteria using a bottom-up approach that involves the enzymatic polymerization of glucose. The diameter size for CNF and BNC are usually below than 100 nm [66]. The morphological properties of BNC are generally similar to those of CNF.

Nanocelluloses combine important cellulose properties such as hydrophilicity and crystalline characteristics, while containing a broad chemical modification capacity and a high surface area. The use of nanocellulose instead of other materials in composites is known to make certain products more efficient, biocompatible, cost-effective and environmentally friendly. Nanocellulose displays much merit thus making it a good material of choice for the development of chemical sensors. 

Table 1 highlights major nanocellulose attributes that contribute to their use as chemical sensors.

As was discussed above, nanocellulose in its native form has limitations in its application as a chemical sensor. Composites of nanocellulose for use as chemical sensors can be prepared through surface functionalization of the nanocellulose with other conducting polymers. A developed porous nanostructure conducting polymer with a large specific surface area that possesses electrical properties is predicted to be an excellent sensing mediator. The electrostatic interactions between the different charges found in nanocellulose composites and the analytes play an important role in the fabrication of ion exchange and permselective membranes. These developed nanocellulose composites can be modified by changing their surface functionality and permselective properties.

**Figure 5:**
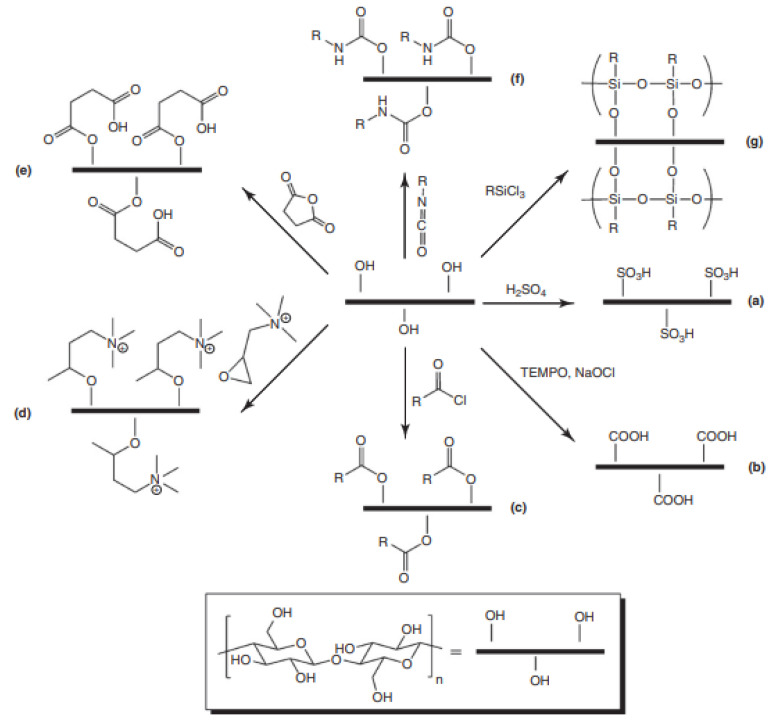
Some possible routes for chemical modification of nanocellulose: (**a**) sulfonation; (**b**) oxidation by TEMPO; (**c**) ester linkages via acid chlorides; (**d**) cationization via epoxides; (**e**) ester linkages via acid anhydrides; (**f**) urethane linkages via isocyanates; and (**g**) silylation. Adapted from Ref. [79].

There are several different strategies of surface functionalization that can be used which involve the chemistry of hydroxyl functional groups found in nanocellulose as shown in Figure 5. Functionalization of nanocellulose can usually be carried out through several reactions such as covalent, oxidation, esterification and even more [77,78]. In order to covalently modify superficial porous nanocellulose, this would involve its treatment with strong acids, silylation, the addition of small functional groups, medium-sized molecules, macromolecules, polymers or even nanoparticles. Besides that, oxidants can also be used for tuning the surface (charges, functional groups) and their properties. TEMPO-mediated oxidation is the most employed reaction to enrich a nanocellulose surface with carboxylic acids. Functionalization of nanocellulose surfaces can also involve esterification reactions using a wide variety of modalities such as acetylation, acylation and cationization, which causes the surface of the nanocellulose to change dramatically through these reactions thereby causing them to possess more hydrophobic or cationic charges.

Additionally, functionalization using other molecules onto nanocellulose surfaces has been extensively described [72,77,78,80,81]. Another very relevant example of surface modification involves polymer-grafting methods onto the nanocellulose. However, a common limitation using this reaction is when both polymer types are incompatible. This becomes a big challenge to increase the polar character of the nanocellulose to enable it to become compatible with other polymers. This incompatibility problem involving covalent linkages can be improved using several techniques. These include “grafting onto” which involves the attachment of an existing polymer onto nanocellulose using coupling agents which can be by employing a polymerization process that uses particular monomers and through the use of an initiator agent in the presence of the nanocellulose during processing. Beyond these, freeze-drying, hot-pressing and casting techniques have also been found to be suitable for increasing the compatibility of nanocellulose within polymeric matrices. However, the problem of nanocellulose agglomeration is that it then promotes sedimentation, which especially in a non-polar matrix continues to persist. Among methods to overcome this, melt extrusion techniques have been used to reduce this agglomeration.

## 4. Chemical Sensor Characteristics and Their Basic Mechanism

**Figure 6:**
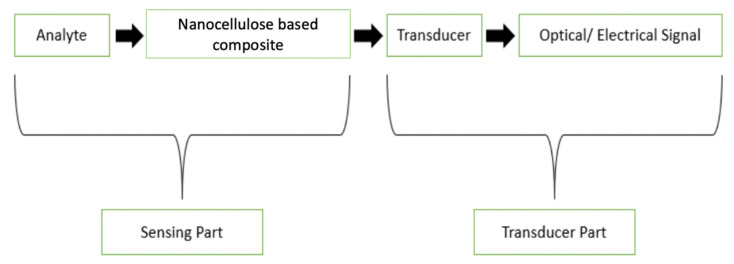
Structure of nanocellulose composites chemical sensors.

A chemical sensor is a device that converts the chemical properties of an analyte into a quantifiable signal. Typically, the analyte concentration is proportional to the magnitude of the signal measured. Chemical sensors are made up of a sensing material and a transducer. The typical manner in which a chemical sensor functions is shown in Figure 6, where once the tested analyte is absorbed by the sensing material, the transducer translates the recognition and sensing events acquired by the sensing material into a measured signal such as conductance, resistance or optical responses [68]. Due to the typically miniscule size of the sensing material, only small quantities of analytes are necessary to modify the electrical characteristics of the sensing material, thus allowing remarkable sensitivity and selectivity of these sensors. This functionality is even able to identify individual molecules and analytes in vapour form. Breath analysers, electrochemical gas sensors, and fiber-optic sensors are a few examples of chemical sensors already developed and in commercial use. In general, chemical sensors can be classified according to the type of analyte to be examined, encompassing gas, biosensor, humidity, optical, and ion sensor types [82]. These sensors can be further classified based upon their working principles, some examples of which are semiconductor, contact combustion, photometric, photochemical and electrochemical types. They are commonly used to precisely determine analyte concentrations or changes occurring in chemical processes. 

The selection of an appropriate substrate and sensing material is critical in order to optimise sensor performance. A further important element when determining a chemical sensor’s best performance is in the choice of its sensing principle. Table 2 shows various different types of sensing principles and the signal/magnitude provided by these principles. As a result of the factors described earlier, researchers in this area need to focus their efforts so as to achieve (1) sensitivities equivalent to those obtained when working with rigid substrates at low or room level sensor working temperatures, (2) high selectivity for the target analyte, (3) fast response and recovery of the sensor, (4) reliable repeatability and stability and (5) sensor constructs that may be bent and/or stretched without permanent damage [83].

Recently, substantial research has been conducted using nanocellulose as a chemical sensor. With the commencement of large-scale nanocellulose extraction [66], researchers all over the globe have used nanocellulose to detect a variety of different analytes. In order to address the key factors stated above, several techniques have been utilised which include sensor material doping, functionalization, hybridization, and deposition [92,93,94]. Chemical sensor mechanisms can be further classified into two types: physisorption and chemisorption. Chemisorption involves both reversible and irreversible reactions which would occur on the sensing surface at the same time. These two forms of reaction would have different rates of adsorption, while having different active sites available with their corresponding activation energies. The rate of physisorption is quicker than the rate of chemisorption during the introduction of a gas and at the early phase of adsorption. However, when more of the available sites become saturated with the analyte, when no reversible sites are able to adsorb analytes, the process then slows down. This differs from the earlier phase pre-saturation, when the sensor responses are directly influenced by the availability of both irreversible and reversible sites. While physisorption and chemisorption does occur simultaneously, physisorption takes precedence over the latter, thereby providing both faster reaction and recovery times [95]. 

**Figure 7:**
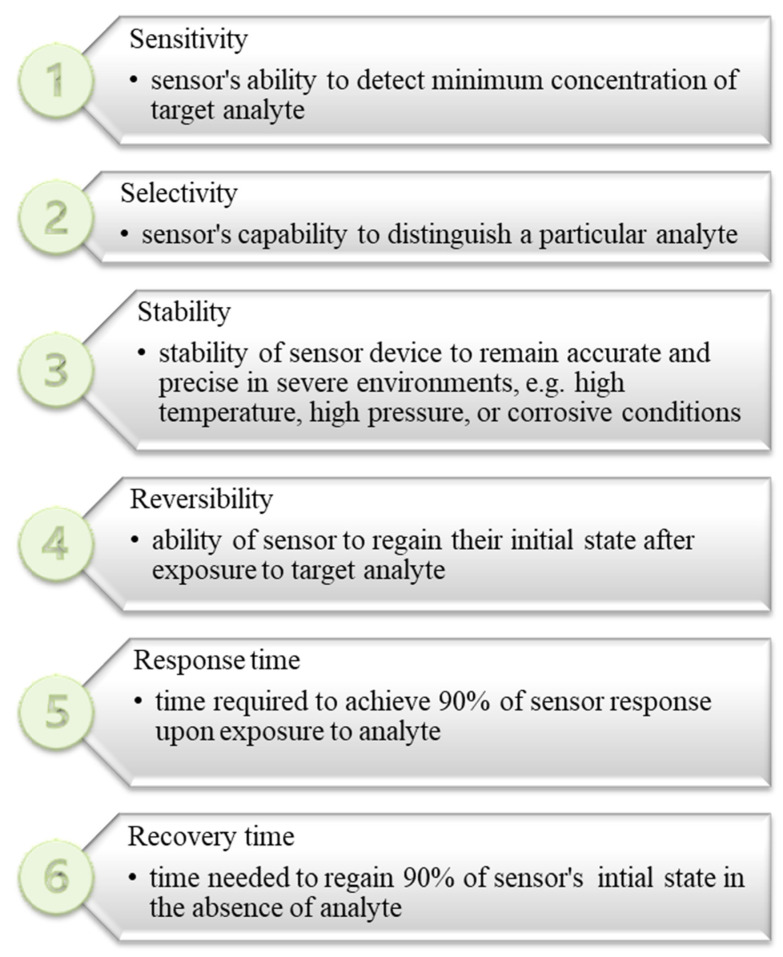
Key components in chemical sensors.

The factors that are of importance in evaluating chemical sensor performance are sensitivity, selectivity, stability, reversibility, response time and recovery time and these are shown in Figure 7 which contains descriptions of the terminology used.

The detection strategies used for chemical sensing are aimed at addressing certain key characteristics of function and to improve sensing performance. The common chemical sensing technologies in use utilise the following mechanisms and principles of function. Sensor response, which is often known as sensitivity, is described as follows:

(1) $$S\%=\frac{R_{g}-R_{o}}{R_{o}}\times 100$$

(2) S(dimensionless)=Sg/Sa

where *R_o_* and *R_g_* are sensor output signals in air and target analyte, respectively. The sensor output signal depends on the sensing principle utilised (e.g., capacitance, resistance, conductance, etc.) [9]. For example, in an electrochemical sensor which is based on a redox reaction, the target analyte interacts at the core of sensor device with an electrolyte and two or three electrodes. 

Normally, the configuration of an electrochemical sensor consists of a sensing electrode, reference electrode and counter electrode which are separated by a thin layer of electrolyte and sometimes with hydrophobic membrane as well. This membrane is installed in the sensor device to protect the sensing electrode, to filter unwanted analytes and to manage the amount of analyte that reaches the surface of the sensing material. The analyte usually reacts with the sensing material by means of an oxidation mechanism. For example, the sensing electrode may provide electrons to the cathode for oxygen reduction. The electrolyte within the sensor then permits ionic charges to migrate from one electrode to another. These reactions would vary based on the type of the analyte intended for detection, and because of this the type of material used to make the electrode becomes critical to the sensor’s sensitivity [96].

Chemical sensor responses are commonly determined by the sensor material active layer’s resistivity but could also utilise other response types depending on the measurement done. Resistive type sensors function by means of a change in conductivity in the sensor material in the presence of target analyte. Thus, the interaction between a target analyte and the sensing material would be dependent on adsorption and desorption mechanisms. In the case of resistive sensors with an n-type sensing layer, when air comes into contact with it, oxygen is adsorbed as oxide ions after electrons are absorbed on the sensing layer surface. When a reducing (or oxidising) vapour interacts with the sensing layer, the analytes (either as donors or acceptors) interact with these adsorbed oxygen ions, thus decreasing (or raising) the electron density in the conduction band. As a result, the sensor’s resistance increases (or reduces) substantially thereby creating the response to be measured. In the case of a p-type sensing layer, the contrary phenomenon would occur [9]. The sensing mechanism of resistive sensors are summarised in Table 3.

## 5. Performance of Nanocellulose Composite Chemical Sensors 

The innovative use of nanocellulose in combination with other materials as a chemical sensor has been emphasised and thoroughly studied by various researchers. As has been mentioned earlier, modification by surface functionalization are critical steps in composite processing in order to give nanocellulose these extraordinary capabilities. For instance, Wang et al. (2021) [97] prepared an ionic conductive hydrogel using cationic CNC (CCNC) using an in situ polymerization process for the stabilization of graphitic carbon nitride (g-C_3_N_4_) in the hydrogel. This polymerization leads to the formation of CCNC-g-C_3_N_4_ complexes which are able to demonstrate high sensitivity in the detection of human motion, speech and inhalation actions. The ionic interaction between the g-C_3_N_4_ and CCNC provided adequate sacrificial bonds that highly enabled the stretchability of this hydrogel and would be beneficial in strain sensor applications [97].

**Figure 8:**
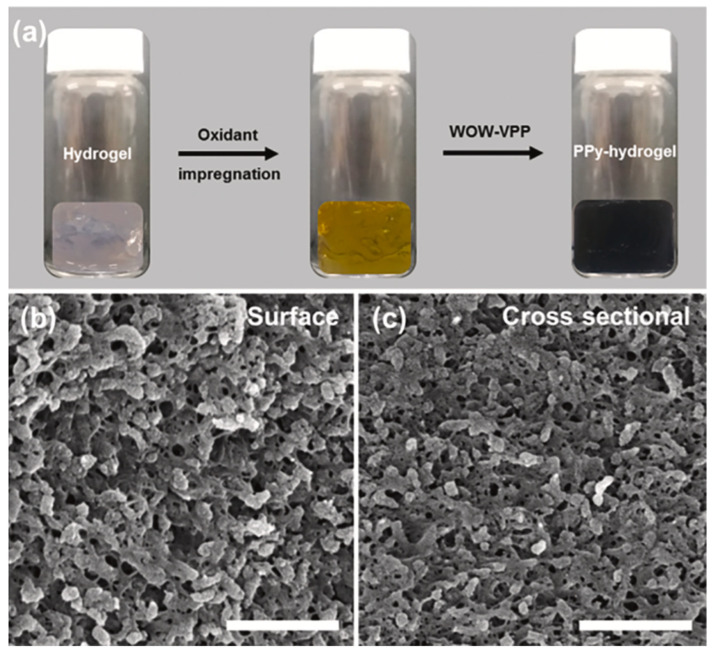
(**a**) Hydrogen of PPy coated on CNC, (**b**) SEM image of the hydrogel surface and (**c**) cross-sectional image of the hydrogel. Reproduced from Ref. [98].

Another different strain sensor was researched by Hanif et al. (2020) [98]. These authors fabricated a hydrogel using CNC with PPy using a wet-on-wet vapor-phase polymerization technique (WOW-VPP). Gelation of CNC can be achieved by addition of polymers in CNC suspensions as done by Hanif et al. (2020) [98]. The oxidant added in the beginning promotes the interaction of PPy in CNC suspension. The adsorption of polymers can lead to bridging at low surface coverage and the formation of CNC gels by stabilization through steric repulsion at high surface coverage. The hydrogel-based strain sensor revealed enhanced resistivity changes under various applied loads and good performance under repeated compression cycles. Typically, vapor phase polymerization involves coating the substrate with an oxidant and this is followed by a short drying period to remove any excess solvent, before the PPy polymerization is done onto the oxidant coated surface within a controlled humidity environment. Usually classical vapor phase polymerization leads to bulk polymerization occurring and the presence of impurities however the WOW-VPP technique does overcome the challenges, and results in a lightweight hydrogel which is a renewable and sustainable material, is needing only simple preparation, is capable of modest scale-up production needs, and can be operated at ambient temperature without having the problem of bulk polymerization occurring during synthesis. Figure 8 displays the hydrogel with PPy coated onto CNC and the SEM images of the resulting hydrogel.

Using conductive nanocellulose, Ouyang et al. (2022) [99] designed a textile sensor to track real-time human movement, which included full finger flexion and extension as well as the sensing of subtle swallowing motions. As a supporting fabric for the sensor, silk-polyurethane was chosen to absorb the conductive units of nanocellulose-PPy. This sensor was fabricated using a reversible yarn framework and when tested achieved exceptional sensitivity (Gauge factor = 8.96) and dynamic durability (5000 cycles) in strain sensing and possessed high conductivity. More crucially, the sensor was able to capture movement in both normal and fever temperature environments, as well as the pH response, and this exceptional performance has since progressed into significant application prospects in human–machine interaction, human health and other related fields. 

Silva et al. (2020) [100], on the other hand, utilized BNC to produce a biomarker for uric acid, 17-estradiol, Pb^2+^, and Cd^2+^ detection in both sweat and urine. In order to be used as a bio-substrate, screen-printed carbon electrodes (SPCE) were coated with BNC. Due to the sensitive nature of the biosensor which was composed of thermoplastic resin, conductive carbon particles with a solid content of 32 wt%, and a solvent, a carbon type electrode was chosen. The SPCE sensor was found to be able to detect hazardous metals such as cadmium (Cd^2+^) and lead (Pb^2+^) effectively with detection limits being 1.01 and 0.43 µM, respectively, which was adequate for the monitoring of these metal ions in human sweat and urine. This functionalized SPCE also managed to detect uric acid and 17β-estradiol with a limit of detection 1.8 μM and 0.58 μM, respectively. The BNC created oxygen groups on the surface of the electrode which improved its wettability and hydrophilicity. Thus these findings show that greener, and more environmentally friendly substrates like nanocellulose do offer good potential for use as immunosensing devices. 

Besides that, Shalauddin et al. (2019) [101] performed electrochemical sensor experiments using nanocellulose composites with *f*-multiwalled carbon nanotubes (*f*-MWCNT) to detect diclofenac sodium (DCF), a non-steroidal anti-inflammatory drug (NSAID) which is a widely used electroactive analgesic. The abundant hydroxyl groups on the surface of nanocellulose provides more binding sites for the adsorption of analytes. *f*-MWCNT provides an axial modulus rearrangement, and possesses a large surface area, excellent electrical conductivity and good mechanical strength. The electrodes fabricated in this study demonstrated two linear dynamic ranges namely, from 0.05 to 1.00 μM and 2–250 μM DCF with a low detection limit of 0.012 μM. In addition, differential pulse voltammetry (DPV) and cyclic voltammetry (CV) profiles done with this sensor displayed remarkable sensitivity and selectivity for the determination of DCF. 

Jung et al. (2017) [102] evaluated the performance of TEMPO-oxidized nanocellulose as both a temperature and pressure sensor. For the pressure sensor role, the authors utilized the piezo resistance principle, where the conducting electrode was inkjet printed onto the TEMPO-oxidized nanocellulose. Experimental data revealed that the sensor was capable of high sensitivity over a wide range (500 Pa–3 kPa) and has high durability over 104 loading/unloading cycles. Whereas for the temperature sensor role, it was modified using a few material types namely, poly(3,4-ethylenedioxythiophene)–poly(styrenesulfonate) (PEDOT:PSS), silver nanoparticles (AgNPs) and carbon nanotubes to form a thermocouple on the upper nanocellulose layer. From the experimental data it was noted that the thermoelectric-based temperature sensors produced a thermoelectric voltage output of 1.7 mV over a temperature difference of 125 K. There findings thus demonstrating that this tactile sensor possessed durable sensing ability, showed quick responses and experienced negligible interference which would be beneficial in flexible electronic sensing applications.

**Scheme 1:**
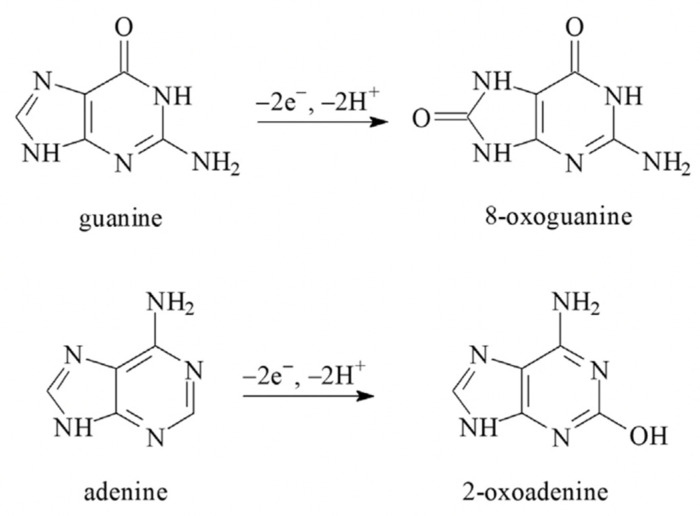
Proposed mechanism of oxidation of guanine and adenine. Reproduced from Ref. [104].

Meanwhile, Ortolani et al. (2018) [103] investigated a sensor fabricated from the functionalization of nanocellulose with single-walled carbon nanohorns (SWCNH) for use in the selective detection of DNA bases. DNA can be measured through the analysis of its bases, and since the analysis of the DNA bases guanine and adenine provides sufficient information for interpretation of DNA sequence, oxidative damage towards DNA, effects of hybridization and protein metabolism in cells, among others, the ability to determine guanidine and adenine is a matter of importance [104]. The developed sensor comprised three electrochemical electrodes which were a platinum electrode as the counter electrode, Ag/AgCl as the reference electrode and a glassy carbon electrode as the working electrode. The working electrode was then immersed into a solution of nanocellulose and single-walled carbon nanohorns within an ultrasonication water bath for few hours. Following this functionalization, it was found that the sensor’s cyclic voltammetry profiles depicted a cathodic direction where the charge transfer process was irreversible for both the analytes. The mechanism of the oxidation of guanine and adenine in this study is represented in Scheme 1. The modified electrode in this sensor displayed lower limits of detection as compared with other electrodes already used for the simultaneous detection of purines. The compatibility seen between nanocellulose and SWCNH gave good electrocatalytic effects and showed great conductivity. The simple preparation and excellent repeatability of this modified electrode indicates huge potential for the determination of guanine and adenine in biosensor applications. 

Sobhan et al. (2019) [105] evaluated the biosensing characteristics of nanocellulose functionalized with activated carbon produced using a casting approach. This nanocellulose composite biosensor was then monitored for its electrical conductivity, water absorption capacity, solubility in water, and its mechanical characteristics were assessed. According to the results, its electrical conductivity decreased as the concentration of nanocellulose increased and this was due to the acquisition of negative charges from its non-conductive components. The composite biosensor film with a nanocellulose composition of less than 50% was noted as ideal for sensor applications. The composite’s tensile strength, strain, and Young’s modulus rose dramatically from 0.03 to 4.78 MPa, 0.13 to 1.94%, and 97.64 to 247.3 MPa, respectively, when the nanocellulose content increased. Thermal decomposition of the composite was noted to occur in the range of 300–400 °C as seen in the thermogravimetric analysis results. The extraordinary biosensing properties of these nanocellulose/activated carbon composites showed promising potential as biosensors for smart food packaging applications. 

Razalli et al. (2017) [106] established an in situ oxidative polymerization method to fabricate PANI and CNC. Following this fabrication, the electrochemical, thermal, structural and morphological properties of the nanocomposite were characterized. The EIS measurements revealed that the PANI/CNC-modified electrode with a smaller semicircle and charge transfer resistance (Rct) value had greater electron transport. The existence of the emeraldine salt of PANI and the inclusion of nanocellulose in the nanocomposite were demonstrated through X-ray diffractometer (XRD) data. Thermal gravimetry analysis (TGA) revealed that high aniline content percentages had inferior thermal stability compared to pure aniline. This study concluded that the developed conductive nanocomposite showed promise for use in sensors, batteries, and conductive adhesives. 

**Figure 9:**
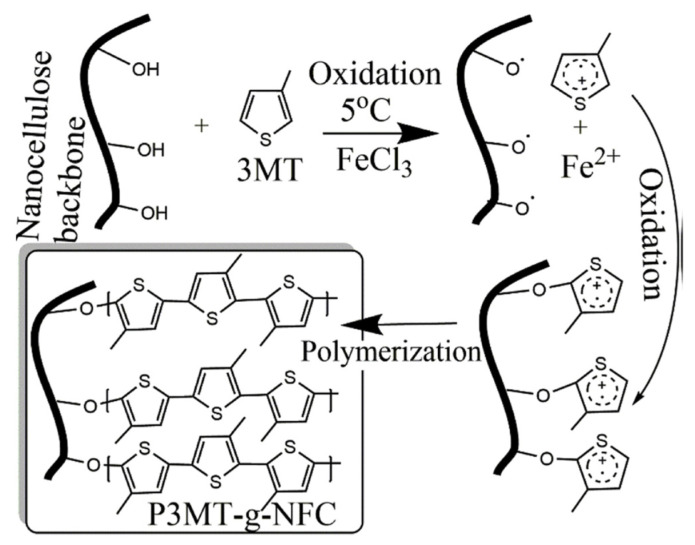
Grafting and polymerization mechanism between polythiophene and CNF. Reproduced from Ref. [108].

Dias et al. (2019) [107] studied the electrical characteristics of CNF modified with polythiophene, a conductive polymer. The reaction time for oxidative polymerization by 3-methyl thiophene monomer on CNF backbones was varied at 2 h, 8 h and 24 h, respectively. Results revealed that a significant amount of conduction polymers was grafted onto the CNF and this was dependent on the polymerization time employed. A longer polymerization time achieved a higher yield of CNF composites. Upon grafting with polythiophene, the insulator characteristics of CNF was converted to conductive and was also enhanced by one order of magnitude when compared to silicon. The oxidative polymerization of CNF also showed exceptional thermal stability, implying that it had potential as a flexible electronic application, particularly as a chemical sensor. Figure 9 illustrates the grafting and polymerization mechanism of CNF and polythiophene.

On the other hand, composites of nanocelluloses with metal nanoparticles (Au or Ag NPs) also have been developed as substrates for surface enhanced Raman scattering (SERS) sensing [108]. For instance, Fu et al. (2022) [109] constructed a flexible 2D nanocellulose-based SERS substrate for pesticide residue detection. The authors successfully developed SERS substrate based on nanocrystalline cellulose (CNC) film via self-assembly of two plasma nanoparticles: gold nanoflowers (AuNFs) and silver-coated gold nanocubes (Au@AgNCs). Pesticide residues on the apple surface could be detected by CNC/Au@AgNC flexible two-dimensional substrate with limit of detection of dimethoate and acetamiprid, 4.1 and 10.7 mg/L, respectively. The precise control of nanoparticle distribution density and uniformity on CNC film contributes to the high sensitivity and field detection of dangerous pesticide residue in a real environment. Song et al. (2021) [110] also fabricated a flexible nanocellulose-based SERS substrate deposited on Au nanoparticles to detect toxic fungicide, thiram. A spiral scanning system was utilized using this flexible and durable nanocellulose-based SERS substrate was provenly sensitive in detection of thiram with detection within 30 s. It is also noteworthy that the error of spiral scanning measurements is smaller than that of multi-spot sampling.

Table 4 tabulates certain other findings on the performance of nanocellulose composite chemical sensors.

## 6. Performance of Nanocellulose Hybrid Composite Chemical Sensors

The development of highly sensitive and selective chemical sensors based on nanocellulose and their polymer hybrid composites has been seen as having significant potential especially in the context of the aforementioned properties. This section will review recent progress in the use of conductive nanocellulose hybrid composites for use in chemical sensors. Differing from the nanocellulose composites discussed in the previous section, the hybrid composites discussed here are those composites which contain a combination of two or more components.

**Figure 10:**
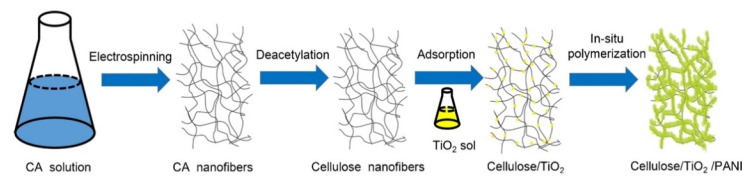
Production steps for CNF/TiO_2_/PANI composite. Reproduced from Ref. [116].

In 2016, Pang et al. (2016) [116] had found that nanofibers’ ammonia sensing characteristics could be improved by the presence of P–N heterojunctions. Deacetylated electrospun cellulose acetate was used to make regenerated CNF, which was subsequently immersed in a TiO_2_ solution to adsorb TiO_2_ nanoparticles onto its surfaces, resulting in a CNF/TiO_2_ composite. To deposit PANI onto the surface of this CNF/TiO_2_ composite, in situ aniline polymerization was applied. The production steps used are depicted in Figure 10.

**Figure 11:**
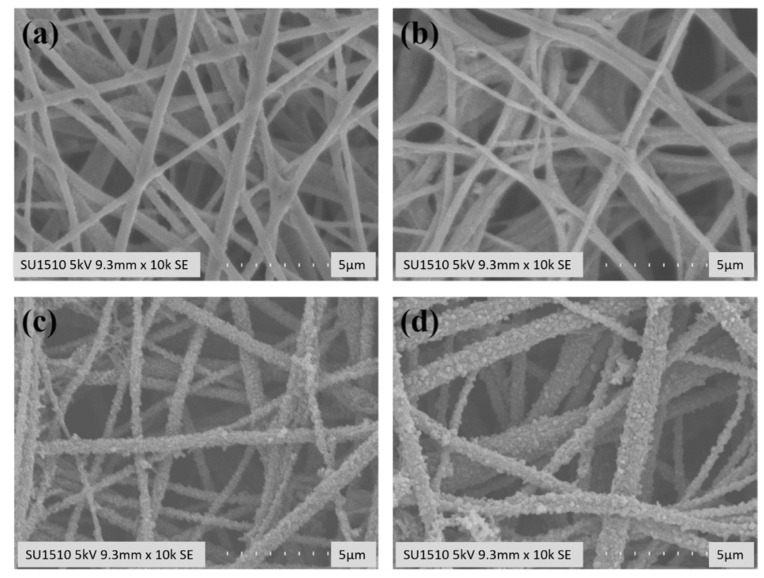
SEM images of CNF (**a**), CNF/TiO_2_ (**b**), CNF/PANI (**c**) and CNF/TiO_2_/PANI composite (**d**). Reproduced from Ref. [117].

**Figure 12:**
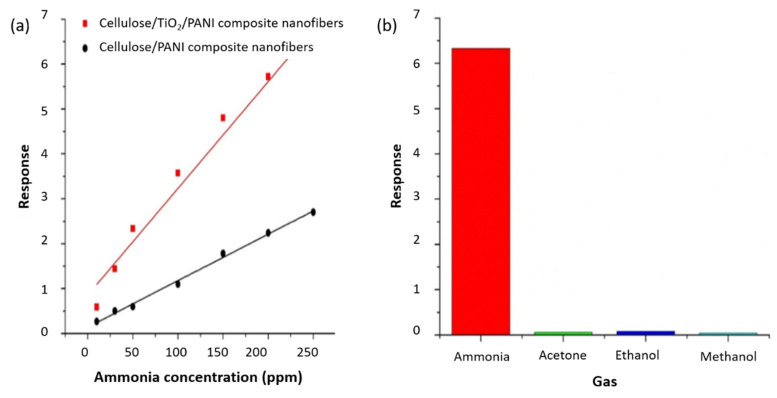
(**a**) Response of CNF/PANI and CNF/TiO_2_/PANI to various concentrations of ammonia (10–250 ppm), (**b**) Selectivity of CNF/TiO_2_/PANI in different solvent gases. Reproduced from Ref. [116].

From the SEM analysis performed (Figure 11b), the surface of the CNF/TiO_2_ was not as smooth as that of CNF (Figure 11a), as is clearly seen in the images below. This could be the effect of TiO_2_ nanoparticle adsorption. The composite with and without TiO_2_ nanoparticles is shown in Figure 11c,d. The surface of the composite became rough due to the presence of PANI. However, the composite maintained a good fiber structure with a greater specific surface area, allowing ammonia vapour migration and composite exposure to the target gases. The response values and sensitivity of this CNF/TiO_2_/PANI composite was found to be substantially greater than those of CNF/PANI alone. The fabricated sensors also showed high selectivity to ammonia gas (Figure 12).

**Figure 13:**
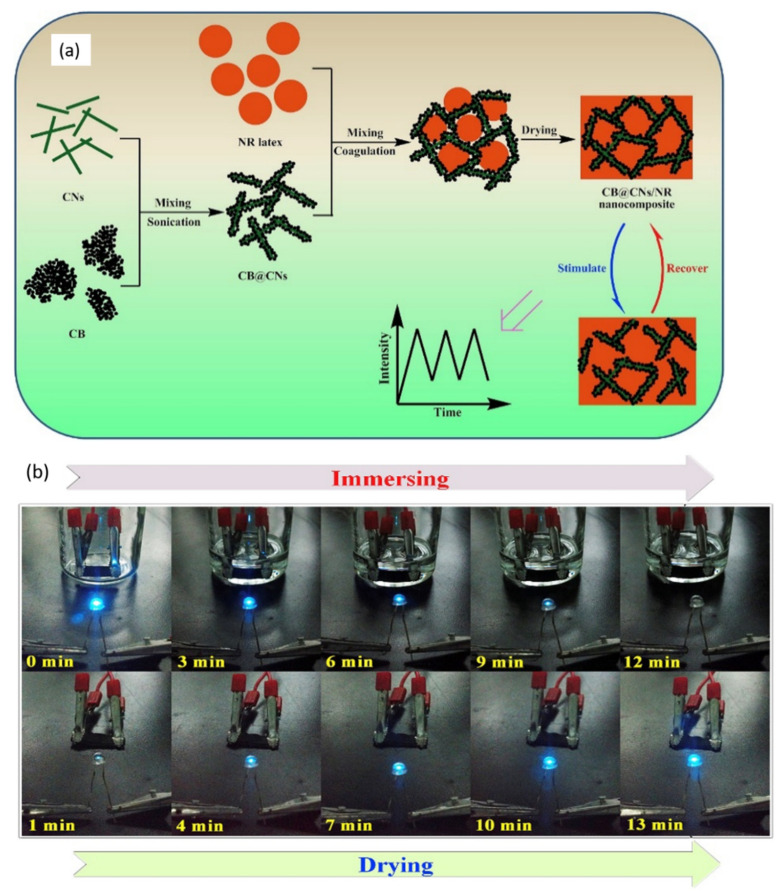

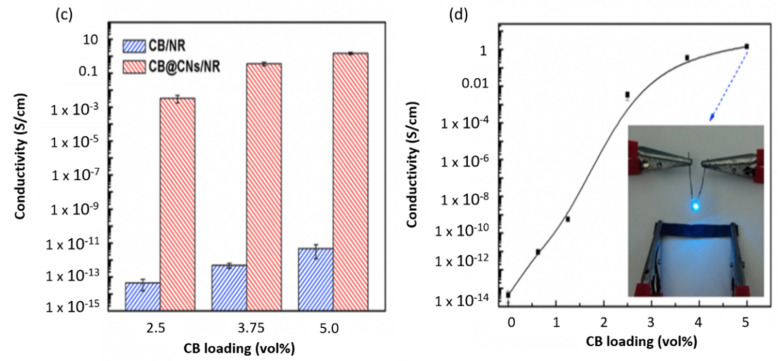
(**a**) Schematic presentation of 3D hierarchical conductive CB/CNC/NR fabrication. (**b**) Images of illumination change during CB/CNC/NR composite (5 vol% CB) immersion into toluene and drying by hot-air. Electrical conductivity responses of CB/CNC/NR and CB/NR with different volume fractions of CB (**c**), electrical conductivity of CB/CNC/NR as a function of CB content (vol%), (**d**) image of CB/CNC/NR lighting up an LED device by 9 V voltage (inset image). Reproduced from Ref. [117].

Besides that, CNC can also be used to make elastic sensors by incorporation into elastomers. In a study done by Wu et al. (2016) [117], CNC was combined with carbon black (CB) and natural rubber (NR) to create a CB/CNC/NR hybrid composite for toluene detection (Figure 13). The study findings revealed that CNC served as a good foundation for CB and directed CB alignment along its axis. Due to the high aspect ratio of CNC, the CB/CNC system displayed colloidal properties with a lower percolation threshold. The electrical conductivity of CB/CNC/NR was 12 orders of magnitude higher than that of CB/NR with the same amount of CB due to this low percolation threshold. The electrical conductivity of CB/CNC/NR was drastically lowered when immersed in toluene due to the swelling of NR in toluene. The percolated network was disrupted and conductivity dropped as the network was diluted in toluene. This was a reversible process, and the network rebuilt after drying to regain its electrical conductivity. The reaction time, responsivity (i.e., resistance variation), and conductivity of the CB/CNC/NR system was found capable of being tweaked by changing the concentration of CB. For instance, at 3.4% CB, the response time to toluene was 168 s, its responsivity was 4427 Ω and its electrical conductivity was 0.35 S m^−1^.

**Figure 14:**
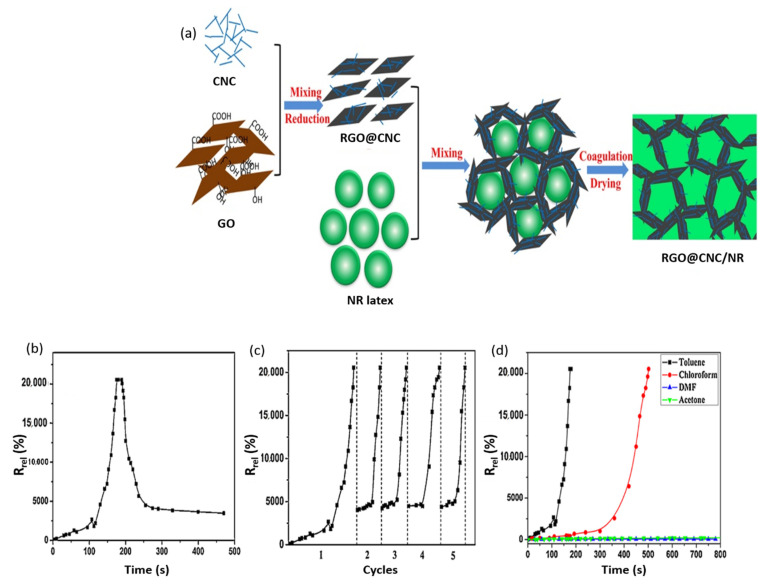
(**a**) Schematic illustration of RGO/CNC/NR with a 3D conductive network. (**b**) Responsivity–time relationship for RGO 2.08 vol% RGO/CNC/NR (**b**) when exposed to toluene the first time, (**c**) during five immersion-drying cycles and (**d**) when exposed to various interfering compounds. Reproduced from Ref. [118].

On the other hand, CNC has also been used to facilitate the assembly of graphene in rubber composites for the chemical sensing of toluene. According to Cao et al. (2016) [118], a composite of reduced graphene oxide (RGO)/CNC/NR, as shown in Figure 14a, had a substantially lower electrical conductivity percolation threshold (0.66 vol%) than RGO/NR after CNC were introduced (1.17 vol%). Furthermore, with the same 0.83 vol% RGO loading, RGO/CNC/NR demonstrated exceptional conductivity, which was 10 orders of magnitude higher than RGO/NR. For organic liquid detection, enhanced RGO/CNC/NR’s conductivity was found advantageous. The sensors were immersed in various organic solvents such as toluene, chloroform, acetone, and dimethyl formamide with 2.08 vol% RGO loading. The resistivity of the sensor was measured over time. Then in the presence of toluene, chloroform, acetone, and dimethyl formamide, relative changes in electrical resistance of the sensor was found to be 2.05 × 10^4^%, 2.05 × 10^4^%, 220%, and 50%, respectively. Despite the fact that the resistance levels of toluene and chloroform were comparable, the sensor’s response time to toluene was significantly faster than that of chloroform (120 s vs. 360 s). Even in the presence of interferences such as chloroform, acetone, and dimethyl formamide, the RGO/CNC/NR remained very sensitive and responsive in detecting toluene during the five immersion-drying cycles (Figure 14b).

**Figure 15:**
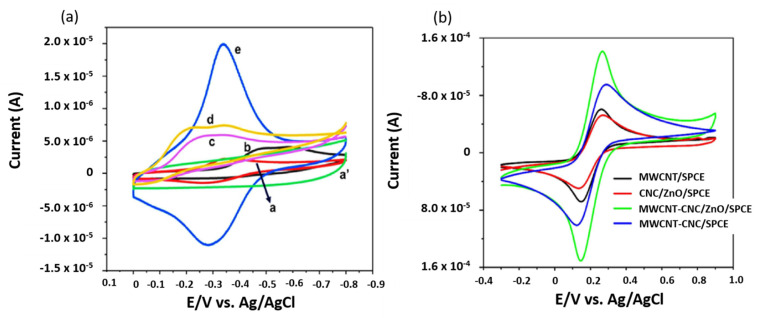
(**a**) Cyclic voltammogram obtained for hemin immobilized different modified SPCEs, hemin/SPCE (**a**) CNC/ZnO NR/hemin/SPCE, (**b**) MWCNT/hemin/SPCE, (**c**) MWCNT-CNC/hemin/SPCE (**d**) and MWCNT-CNC/ZnO NR/hemin/SPCE (**e**) in a deoxygenated pH 7.0 at a scan rate of 100 mV/s. Under the same conditions, MWCNT-CNC/ZnO NR modified SPCE in the absence of hemin (**a′**), (**b**) Cyclic voltammetric response and the electron transfer characteristics of MWCNT-CNC/ZnO NR, CNC/ZnO, MWCNT-CNC and pristine MWCNT modified SPCEs in 5 mM potassium ferricyanide containing 0.1 M KCl. Adapted from Ref. [119].

In the year 2020, Selvakumar et al. (2020) [119] produced a simple solution based on sonochemical synthesis of the novel composite MWCNT-CNC/ZnO NR/hemin. The MWCNT-CNC/ZnO NR/hemin electrode, as constructed, was also employed as an improved sensor matrix for H_2_O_2_ electrochemical measurement in real time. The MWCNT-CNC/ZnO NR/hemin modified screen-printed carbon electrode (SPCEs) electrochemical redox behaviour and electron-transfer properties were compared. The results showed that hemin/MWCNT and hemin/CNC-ZnO demonstrated 1.5 and 2.3 times higher electrocatalytic activity respectively with a lower reduction potential (0.2 V) towards H_2_O_2_ than other examined hemin modified electrodes. In a linear concentration of H_2_O_2_ solution ranging up to 4183.3 M, the constructed sensor demonstrated a consistent amperometric response (−0.2 V vs. Ag/AgCl) with a lower detection limit of 4.0 nM (Figure 15).

Moreover, Mahmoud et al. (2020) [120] modified a sensing electrode using nanocellulose, nitrogen and sulphur co-doped graphene quantum dots for the electrochemical detection of olanzapine (OLZ), an atypical antipsychotic used to treat schizophrenia and bipolar disorders. Polar groups on the surface of the nanocellulose provided additional anchoring sites for the OLZ at the working electrode. Thus, this modified electrode was found to promote excellent selectivity, as well as reliability towards OLZ detection.

Tanguy et al. (2021) [121] presented lignocellulosic CNF (LCNFs) as a renewable and strong substrate for the fabrication of highly sensitive and selective sensors based on PANI and reduced graphene oxide (rGO). The hydrophobic lignin covalently bonds to cellulose in the nanofibrils, thus increasing the water resistance of the sensor and limiting the effects of humidity on the sensor. PANI was deposited on the LCNFs and rGO by means of chemical oxidative polymerization and this composite then showed improvement in its sensitivity to detect volatile amines while still maintaining an acceptable selectivity. The detection mechanism for volatile amines is as follows: (1) adsorption of the analytes onto the sensing layer (PANI backbone), (2) ammonia molecules convert to ammonium cations via acid–base interactions, (3) ammonium cations then immobilize the polarons on the PANI backbone and (4) lastly, mobility and amount of carrier concentration reduces and hence, this increases the sensor’s resistance. The PANI sensor acts as p-type semiconductor, and the presence of rGO magnifies the magnitude of the resistance changes seen. After removal of the analytes, the sensor was found to be able to regain back its initial state which shows the ability for good reversibility of the sensor. Therefore, LCNFs show excellent potential as utilising renewable resources, possessing mechanical strength and forming a strong substrate for use in the fabrication of both sensitive and selective volatile amine sensors.

Table 5 tabulates certain other findings regarding the performance of nanocellulose hybrid composite chemical sensors.

## 7. Challenges and Future Directions

This review discussed an overview of how nanocellulose composites have been designed and fabricated into chemical sensors in order to detect certain analytes. Overall, these fabricated chemical sensors utilizing nanocellulose with its high surface area or porous structure, allows mass transport between the analyte and immobilized indicators in a faster manner and subsequently this translates into response times that can be expected to be significantly shorter. 

However, there some challenges and future directions related to this research that need to be taken into consideration and need to be addressed, as follows:

(a) Agglomeration is a major issue that must be taken into account when preparing nanocellulose for use as this is a result of nanocellulose’s inherent large surface area properties. It should be emphasized that aggregated nanocellulose is very difficult to be re-dispersed, which can lead to undesirable effects on sensing abilities and mechanical properties of the resultant composites. Due to its hydrophilic properties, nanocellulose is also difficult to dry, becomes prone to aggregation and is incompatible with other hydrophobic materials.
(b) Functionalization is an important key towards success in order to make nanocellulose a conducting polymer composite. There is still gaps in knowledge of the crystallinity and surface properties of functionalized nanocellulose, which can have a significant impact on the fabrication, reproducibility and applications of nanocellulose composites within chemical sensors. Methods such as nuclear magnetic resonance spectroscopy can be used to determine the chemical structure of these materials. This functionalization has been a key factor in many of the sensor technologies described in this review. Many of the sensor technologies highlighted have shown high sensitivity and selectivity and low cost, which are essential attributes for their commercialization and wider use in the industry.
(c) Pretreatment has been a feature in a number of the developments described earlier. The development of compact and portable nanocellulose composite chemical sensors which require less complicated pretreatment steps is a matter that needs attention to capitalise on the advantages that nanocellulose composites possess to enable better and more efficient sensors to be made.
(d) Major hurdles for the commercial application of nanocellulose lies both in their processes of fabrication and post-production preparation. Green nanocellulose extraction approaches and functionalization techniques that minimize acid and solvent wastes are needed. Moreover, the presence of chemical contaminants arising from functionalization processes must be avoided
(e) Research aimed at optimizing the dispersion and orientation of nanocellulose within or on composite matrices needs further exploration. This is an important key towards structural integrity and sensing performance of these sensors. There have been several studies exploring the use of a variety of different compatibilizer in polymer composites of nanocellulose aimed at improving the dispersion and arrangement of fibers in these composites.

Discoveries regarding the functionalization of nanocellulose with other different nanomaterials is still very much in its early stages. There are several other types of nanomaterials that could potentially be good conducting polymers capable of enhancing the properties in nanocellulose composites used in sensing applications. Thus, scientists still have a nascent research area to discover greater potentials for the functionalization of nanocellulose with other nanomaterials in order to create newer, more efficient and yet more environmentally friendly sensors for a multitude of applications.

## Figures and Tables

**Table 1:** Merits of nanocellulose as chemical sensors.

Properties	Description	References
Surface modification	- Through surface modifications of the nanocellulose and its permselective properties, this allows the modification of a new membrane for different species. - The high energy interaction between binding sites on the nanocellulose film and cationic species when combined with its permselective properties make the nanocellulose film a prime candidate for sensor devices.	[67,68]
Nanocellulose structuring in solid films	- Orientation and alignment of nanocellulose is important in the development of new chemical sensors. Nanocellulose is very suitable for this as it forms ordered structures. - Among the several types of nanocellulose, CNC is of special interest in these applications because of its inherent characteristics relating to aspect ratio, cylindrical shape, rigidity and chiral ordering, all of which lead to desired optical effects in aqueous media, in films or in solids when templated from these structures.	[69,70]
Large specific surface area/porous structure	- The response time in a sensor which is based on a bulk polymer is relatively long due to slow penetration of the target molecules into the polymer. - However, because of the porous structure of nanocellulose, this response time is expected to be significantly faster. - The sensitivity and response time of a sensor is highly dependent on the specific surface area of the mediator chosen. The target analyte must be able to adsorb onto and diffuse into a mediator which possesses a high surface area and consequently, both higher sensitivity and faster response time can be expected.	[71,72]
Tensile	- Nanocellulose is known to have high tensile strength, therefore, it would be useful in fiber-optic type sensors for strain sensing applications, such as in Fiber Bragg Gratings, helical long-period fiber gratings, and Fabry–Perot interferometers.	[73]
Optical/fluorescent	- The fluorescent properties of nanocellulose are also one of the chemical modifications that can be used to develop sensing materials. - The use of analyte-sensitive fluorophores along with a reference fluorophore that can react with the hydroxyl groups in nanocellulose will allow ratiometric measurements of an analyte.	[74,75]
Electrical	- Nanocellulose can be chemically modified to make it a good electrical conductor with permselective properties.	[74]
Biodegradable	- The biodegradable properties of nanocellulose are useful for development into chemical sensors which are appropriate in medical implants, environmental sensors, and as wearable and disposable devices.	[76]

**Table 2:** Different types of sensing principles and signals provided by these principles.

Sensing Principles	Magnitude or Signal	Example of Sensing Material	Advantages	References
Chemiresistive	Resistance of sensing layer	- Carbon nanotubes - Metal-oxide semiconductor - Graphene nanoparticles	- Presence of analyte easily measured - Simple sensing mechanism	[84]
Capacitive	Change of dielectric constant of sensing layer at high frequency	- Copper - Indium tin oxide - Printed ink	- More responsive than resistive touchscreen - Detects solid and liquid analytes without physical contact	[85]
Impedance metric	Change of impedance of sensing layer at high frequency	- Graphene - Metallic nanowire - Molybdenum disulphide	- Non-invasive and label-free analysis - Wide linear range of responses	[86]
Field effect transistor	Change of source-drain current	- Silicon nanowire - Conducting polymer - Metal oxide	- Ultra-high sensitivity sensing, label-free operation, cost reduction and miniaturisation.	[87,88]
Mechanical	Change of wave propagates on the surface of the sensing layer	- Carbon nanotubes - Reduced graphene oxide - PPy	- Detects mechanical deformation such as touch, force, acoustic, inertial and tactile responses	[89]
Optical	Change in the spectrum of the sensing material	- Tin disulfide - Carbon dots - Metal hydrides	- Cost effective and simple hardware setting - Low-power-consumption	[90,91]

**Table 3:** Sensing mechanism of resistive sensors.

Sensing Response Behaviour	Target Analyte	n-Type Sensor	p-Type Sensor
Oxidising analytes	Cl_2_, N_2_O, O_2_, compressed air, H_2_O_2,_ F_2_	Resistance increases	Resistance decreases
Reducing analytes	H_2,_ CO, H_2_S, CH_4,_ NH_3_, C_6_H_6_	Resistance decreases	Resistance increases
Minority carrier	-	Hole (+)	Electron (−)
Majority carrier	-	Electron (−)	Hole (+)

**Table 4:** Performance of nanocellulose composite chemical sensors.

Composite	Target Analyte	Performances	Advantages	References
Silver nanoparticles/BNC	Methomyl	- Limit of detection: 3.6 × 10^−7^ M - Stability: displayed great sensitivity after 3 months	- User-friendly device - Simple and fast results - Portable	[111]
Polyaniline/CNC (screen-printed electrodes with thin layer ionic liquid)	Cholesterol oxidase	- Sensitivity: 35.19 μA mM/cm^−2^ - Dynamic linear range: - 1 μM to 12 mM (R^2^ = 0.99083) - Limit of detection: 0.48 μM - Reproducibility (RSDs ≤ 3.76%) and repeatability (RSDs ≤ 3.31%)	- The developed sensor provides high porosity and good electrocatalytic activity - Great selectivity and stability	[112]
CNF/carbon dots	Glutaraldehyde	- Maximum carbon dot grafting content on CNF aerogel: 113 mg/g - High fluorescence quantum: 26.2% - Mechanical properties: increased to 360% of compression strength	- Carbon dots serve as stabilizers to endow shape recovery in water - Detection at parts per million (ppm) level - High selectivity towards NO_x_ analyte	[113]
Nanocellulose/polyethylene terephthalate (PET)	Glucose oxidase	- Limit of detection: 0.1 × 10^−3^ M - Response time: 8–10 min - Glucose concentration: 3 g L^−1^	- Inexpensive, disposable, and environmentally friendly	[114]
Graphene-nanocellulose (impregnated in polydimethylsiloxane for stretchable nanopaper)	Bending and stretching	- Stretchable: 100% without breaking - Gauge factor: 1.6 at 10% strain to 7.1 at 100% strain	- Efficient detection of a variety forms of forces - Stretchable > 10 times more sensitive than CNT and AgNWs	[115]

**Table 5:** Performance of nanocellulose hybrid composites chemical sensor.

Material	Target Analyte	Performances	Advantages	References
Polydiacetylene/CNC/chitosan	Ammonia	- Presence of negatively charged CNC in the system increased stability of polydiacetylene. - Sensor exhibits distinctive blue-to-red colorimetric response after being exposed to spoiled meat, even at sub-zero temperatures.	- Low cost, highly sensitive sensor. - Can be incorporated into food packaging to monitor food spoilage.	[122]
AgNPs/carboxymethyl cellulose/CNF	Catechol	- Cyclic voltammetry reveals that the developed sensor was of benefit to immobilize Lac and facilitate direct electron transfer between Lac and electrode. - The developed sensor has a detection limit of 1.64 μM (S/N = 3), and a wide linear range from 4.98 μM to 3.65 mM.	- Can be applied as substrate material for different biosensors by immobilizing other different functional nanoparticles or enzymes. - Good repeatability, reproducibility, stability, and selectivity.	[123]
Lac/Ag/ZnO/CNF	Catechol	- Highly sensitive detection of catechol with wide linear range from 0.995 to 811 mM and low detection limit of 0.205 mM.	- Promising nanostructured material for the fabrication of different sensors.	[124]
Peptide/poly(vinyl alcohol)/CNC	Trypsin	- The developed sensor has nearly instantaneous and pronounced change in fluorescence emission intensity in response to changes in pH. - Trypsin detected at a concentration of 250 μg/mL (concentration that is typical for abnormal proteolytic activity in wound fluids).	- A versatile scaffold for the development of new sensors for applications in wound diagnosis and other biomedical applications.	[70]
Graphene oxide/PPy/CNC	Glucose	- The composite exhibits high sensitivity (ca. 0.73 μA·mM^−1^), with high dynamic response ranging from 1.0 to 20 mM glucose. - Exhibits a limit of detection (LOD) of (50 ± 10) μM. - And excludes interfering species, such as ascorbic acid, uric acid, and cholesterol.	- The developed sensor displays an acceptable reproducibility and stability over time. - Current response was maintained over 95% of the initial value after 17 days.	[125]
Poly(diallyldimethyl ammonium chloride/carboxylated CNC/poly(3,4-ethylenedioxythiophene)	Nitrite	- The developed sensor can detect nitrite in the 0.2 μM to 1.73 mM concentration range with a 57 nM detection limit.	- The composite has lowest electrochemical impedance and best electrocatalytic activity towards the oxidation of nitrites.	[126]

## Data Availability

Not applicable.
